# The Nature and Physiological Action of Radium Emanations and Rays

**Published:** 1904-01-09

**Authors:** 


### THE NATURE AND PHYSIOLOGICAL ACTION OF RADIUM EMANATIONS AND RAYS.

DR. DAWSON TURNER,[1] speaking of various forms of radiation which are used in practical medicine and surgery arranges them in five groups:—(1) Radiant heat rays, (2) ordinary light, (3) ultraviolet light, (4) Roentgen rays, (5) radium emanation and rays. Below the ordinary spectrum of white light are the long radiant heat waves, above are the short ultra-violet rays. These latter, with blue indigo and violet constitute the Finsen rays. Their chief properties are that they are (*a*) powerfully actinic, (b) can excite fluorescence, (c) can discharge an electrified body, (d) have clinical and bactericidal effects, (e) can produce cloud nuclei in moist air. Roentgen rays are probably irregular wave impulses, and differ from the ultra-violet light in their power of penetration, their lack of bactericidal effect, and the fact that a severe inflammation and skin destruction may result from their use. Radium gives off a radio-active emanation and three kinds of rays labelled $\alpha$, $\beta$, and $\gamma$. It gives off heat, and is of higher temperature than its surroundings.

The emanation is a luminous gas, which imparts radio-activity to objects in its path. This gas, when breathed, would cause the lining membrane of the bronchi and lungs to be radio-active and bactericidal, and might influence the course of lung disease. But there would probably be considerable danger in this form of treatment. The $\alpha$-rays have little penetrative power. The $\beta$-rays are powerfully actinic, they resemble the cathode rays of a Crooke's tube described by Lenard. The $\gamma$-rays are the most penetrative and resemble $x$-rays. Radium rays have bactericidal properties; they also have influence over the growth of cancerous tumours. It is unsafe to carry radium in the pocket, as inflammation and ulceration of the skin often results, an intractable sore so originating may not heal for months. It has an action on the brain and nervous system, producing paralysis and death. Totally blind people are unaffected by radium, but those who are nearly blind can perceive light when radium is brought near them, and can detect the form of objects brought near the radium screen.

Dr. Macintyre says that the results of radium treatment in cases of malignant disease have so far been rather unsatisfactory. The action of $\gamma$-rays is similar but much less powerful than the ordinary $x$-rays. In lupus the radium emanations and rays have a beneficial action, the lesion being affected in much the same manner as when Finsen rays or high frequency currents are used.

[1] Brit. Med. Jour., Dec. 12.

